# The safety of beta-blocker use in chronic obstructive pulmonary disease patients with respiratory failure in the intensive care unit

**DOI:** 10.1186/2049-6958-9-8

**Published:** 2014-02-04

**Authors:** Feyza Kargin, Huriye Berk Takir, Cuneyt Salturk, Nezihe Ciftaslan Goksenoglu, Can Yucel Karabay, Ozlem Yazicioglu Mocin, Nalan Adiguzel, Gokay Gungor, Merih Kalamanoglu Balci, Murat Yalcinsoy, Ramazan Kargin, Zuhal Karakurt

**Affiliations:** 1 Respiratory and Intensive Care Unit, Sureyyapaşa Chest Diseases and Thoracic Surgery Training and Research Hospital, Soyak Yenişehir Manolya Evleri, 34770 Umraniye, Istanbul, Turkey; 2 Department of Cardiology, Sureyyapaşa Chest Diseases and Thoracic Surgery Training and Research Hospital, Istanbul, Turkey; 3 Department of Cardiology, Koşuyolu Kartal Heart Training and Research Hospital, Istanbul, Turkey

**Keywords:** Acute respiratory failure, Arrhythmia, Beta-blockers, COPD, Intensive care unit

## Abstract

**Background:**

The safety of beta-blockers as a heart rate-limiting drug (HRLD) in patients with acute respiratory failure (ARF) due to chronic obstructive lung disease (COPD) has not been properly assessed in the intensive care unit (ICU) setting. This study aims to compare the use of beta-blocker drugs relative to non-beta-blocker ones in COPD patients with ARF due to heart rate-limiting with respect to length of ICU stay and mortality.

**Methods:**

We performed a retrospective (January 2011-December 2012) case-control study in a level III ICU in a teaching hospital. It was carried out in a closed ICU by the same intensivists. All COPD patients with ARF who were treated with beta-blockers (case group) and non-beta-blocker HRLDs (control group) were included. Their demographics, reason for HRLD, cause of ARF, comorbidities, ICU data including acute physiology and chronic health evaluation (APACHE II) score, type of ventilation, heart rate, and lengths of ICU and hospital stays were collected. The mortality rates in the ICU, the hospital, and over 30 days were also recorded.

**Results:**

We enrolled 188 patients (46 female, n = 74 and n = 114 for the case and control groups, respectively). Reasons for HRLD (case and control group, respectively) were atrial fibrillation (AF, 23% and 50%), and supraventricular tachycardia (SVT, 41.9% and 54.4%). Patients’ characteristics, APACHE II score, heart rate, duration and type of ventilation, and median length of ICU-hospital stay were similar between the groups. The mortality outcomes in the ICU, hospital, and 30 days after discharge in the case and control groups were 17.6% versus 15.8% (p > 0.75); 18.9% versus 19.3% (p > 0.95) and 20% versus 11% (p > 0.47), respectively.

**Conclusions:**

Our results suggest that beta-blocker use for heart rate control in COPD patients with ARF is associated with similar ICU stay length and mortality compared with COPD patients treated with other HRLDs.

## Background

The majority of patients with chronic obstructive pulmonary disease (COPD) have chronic heart failure (CHF) or coronary artery disease (CAD) [1]. The risk of cardiac arrhythmia is increased during acute exacerbations of COPD [1]. Atrial fibrillation (AF) is frequently observed in elderly COPD patients [2], and cardiac arrhythmias are a significant cause of mortality in these patients [3]. COPD patients with CAD generally have elevated heart rates, and beta-adrenergic receptor antagonists (beta-blockers) are known to improve the survival of patients with CHF or CAD [4,5]. Previous studies have shown that patients with CAD and coexisting COPD generally failed to receive optimal therapy or appropriate drug dosages for HR reduction. In a recent study, 54% of patients suffered from heart failure in a population with CAD and COPD, and only 52.8% of these patients were receiving beta-blocker therapy. In addition, in the majority of these patients the daily dosages of beta-blockers were very low, which could be explained by the physicians’ hesitation to prescribe them due to possible adverse pulmonary effects [6]. The current medical treatment approach for COPD exacerbation is the administration of short-acting bronchodilators (ß_2_**-**agonists), with or without anticholinergic compounds and corticosteroids [7]. COPD patients with arrhythmias require complex treatment strategies. Studies comparing the side effects of beta-blockers have yielded mixed results in patients with arrhythmias and myocardial infarction (MI) [8-11].

There are limited data about patients with acute respiratory failure (ARF) in the intensive care unit (ICU) that demonstrated the safety of beta-blockers in COPD patients with arrhythmias. In this study, we compared the outcomes of COPD patients with ARF who received beta-blockers versus other medications for heart rate control. We hypothesized that the use of beta-blockers as a heart rate-limiting drug (HRLD) in COPD patients with ARF would achieve similar outcomes with respect to mortality and length of ICU stay as other non-beta-blocker drugs.

## Methods

We performed a retrospective case–control study in the ICU of a teaching hospital for chest diseases between January 2011 and December 2012. The 22-bed ICU was classified as level III and was operated as a closed unit by eight pulmonologist-intensivist specialists 24 hours/day and 7 days/week. The majority of ARF patients admitted to the ICU had COPD. This study was approved by the local ethical committee of the government teaching hospital (Sureyyapasa Chest Diseases and Thoracic Surgery Teaching Hospital-Istanbul-Turkey).

### Patients

All consecutive patients with previously diagnosed COPD who were admitted to the ICU due to ARF during the study period were evaluated. COPD diagnoses were established by a physician based on airflow obstruction on spirometry, defined as a forced expiratory volume in 1 second (FEV_1_) and forced vital capacity ratio of 70% or less. We searched the electronic database system and International Classification of Disease (ICD)-10 coding system and recorded “J 44” as COPD. Patients were enrolled in the study if they had been treated with HRLDs at any time during their ICU stay. Patients with asthma or previous use with diltiazem were excluded from the study, as were those who had been in the ICU for less than 24 hours. The patients were divided into two groups according to their HRLD treatment: beta-blocker (case group) and non-beta-blocker (control group). The HRLDs administered in the case group included metoprolol, bisoprolol, and carvedilol, and those given to the control group were diltiazem, digoxin, amiodarone, or any combination of these.

### Data

The presence of comorbidities, including diabetes mellitus (E 10–11), ischemic heart disease (I 25), and atrial fibrillation (I 48), was determined by the ICD coding in our teaching hospital database. Previous HRLD use was also recorded at the time of ICU admission. Any previous hospital admissions were recorded as emergency service, hospital ward, and other ICU. Body mass index (BMI), reason for HRLD use, causes of ARF, acute physiological and chronic health evaluation (APACHE II score) [12], application and duration of noninvasive (NIV) or invasive mechanical ventilation (IMV), heart rate, C-reactive protein (CRP) level at ICU admission and the peak value during ICU stay, biochemistry values, complete blood counts, sputum/tracheal aspirate/bronchial lavage cultures, lengths of ICU and hospital stays, and mortality in the ICU and hospital were recorded from the patients’ files. Mortality within 30 days of hospital discharge was recorded from the online deceased declaration system to assess short-term mortality.

### Definitions

#### ARF

Patients were divided into three groups according to their arterial blood gas (ABG) results on admission to the ICU. “Hypoxic ARF” was defined if partial arterial oxygen pressure in inspired fractionated oxygen (PaO_2_/FiO_2_) was < 300 and partial arterial carbon dioxide pressure (PaCO_2_) was < 45 mmHg. “Hypercapnic and hypoxemic ARF” was defined as PaCO_2_ > 45 mmHg and PaO_2_/FiO_2_ < 300, and “hypercapnicARF” was PaCO_2_ > 45 mmHg and PaO_2_/FiO_2_ > 300 [13,14].

#### Sepsis

The presence of sepsis was defined as the presence of infection together with systemic inflammatory response syndrome [15,16]. Patients who were unresponsive to fluid resuscitation and required vasopressor agents were defined as being in septic shock [16].

### Treatment

**HRLDs** used in the ICU were recorded in six major indication subgroups: 1) AF [17], 2) supraventricular tachycardia (SVT) [18], 3) ventricular tachyarrhythmia (VT), 4) congestive heart failure CHF [19], 5) hypertension (HT) [20], and 6) suspicion of MI. These drugs were administered orally or via intravenous (IV) injection or infusion. The drugs and doses for CHF were as follows: metoprolol (12.5/25 mg once daily [o.d.], starting dose targeting 200 mg o.d.) with bisoprolol (1.25 mg o.d., starting dose targeting 10 mg o.d), and carvedilol (3.125 mg, twice daily, starting dose targeting 25-50 mg), or digoxin (0.25 mg o.d.) [19]. The drug doses for AF treatment were metoprolol (2.5-5 mg IV bolus over 2 minutes, maximum 3 doses, oral maintenance dose 100-200 mg, o.d.), bisoprolol (2.5-10 mg, o.d.), carvedilol (3.125-25 mg, twice daily), diltiazem (60 mg twice daily to 360 mg o.d.), digoxin (0.5-1 mg IV, then 0.125-0.5 mg o.d.), or amiodarone (5 mg/kg in 1 hour, 50 mg/hour maintenance; or in oral form, 100-200 mg once daily) [17]. More than one HRLDs (except beta-blockers) were defined as diltiazem plus amiodarone and/or plus digoxin. While using these drugs, the target heart rate was 80-100/minute [17]. No HRLDs were administered if patients had septic shock. The choice of HRLDs was totally dependent on intensivist staff choice unless a known contraindication was present (e.g., diltiazem was not used in patients with lower ejection fractions).

### Treatment of COPD exacerbation

A short acting ß_2_ agonist (salbutamol, 100 mcg per puff) and ipratropium bromide (100 mcg/20 mcg per puff) were given every 2-4 hours (4 to 10 puffs) via a metered dose inhaler chamber (Aerovent, Altech®, Altera Firm, Izmir-Turkey) when the patients were under NIV or IMV. A nebular form of salbutamol (2.5 mg/2.5 mL per nebule) was given every 15 minutes to 4 hours, or ipratropium bromide/salbutamol (0.5 mg/3.01 mg/2.5 mL per nebule) was given every 2 to 4 hours for patients breathing without mechanical ventilation support. Long-acting ß_2_-agonists were not used in COPD patients with ARF in the ICU [7,21]. The long-acting ß_2_-agonists, such as salmeterol and formeterol, were not used during the ICU stay. A systemic corticosteroid, prednisolone (30–40 mg per day), was also administered [22]. Theophylline was not used in eligible tachycardic COPD patients. Antibiotics were administered according to recommended guidelines [22].

### Mechanical ventilation

NIV was applied initially in the respiratory ward by a specialized staff (nurse and specialist or resident) if patients were hemodynamically stable, cooperative, had no organ failure except respiratory failure, their ABG analysis revealed pH 7.28-7.34, PaCO_2_ = 45-90 mmHg, PaO_2_/FiO_2_ > 200, and the Glasgow coma scale was > 13 due to hypercapnic confusion. ICU demand for patients under NIV treatment in the ward was determined based on cognitive function deterioration, lack of cooperation, agitation, hemodynamically unstable conditions (e.g., hypotension, HT, tachycardia, arrhythmia) that needed close monitoring, and deterioration of ABG values (increased PaCO_2_, decreased PaO_2_, and decreased pH level). NIV was provided in pressure assist-control mode with ICU mechanical ventilators via a double-tube circuit with a full-face mask [13]. Invasive mechanical ventilation was applied in the presence of absolute or relative contraindications for NIV and in NIV failure [13]. Contraindications of NIV were defined as: 1) *absolute*, respiratory arrest and unable to fit mask, and 2) *relative*, medically unstable (hypotensive shock, uncontrolled cardiac ischemia or arrhythmia, upper gastrointestinal bleeding), agitation, uncooperativeness, inability to protect airway, impaired swallowing, excessive secretions not managed by secretion clearance techniques, multiple (two or more) organ failure, and recent upper airway or upper gastrointestinal surgery [13,14]. The definition of NIV failure in hypercapnic patients was no pH improvement, no change or a rise in breathing frequency after 1–2 hours, and lack of cooperation. For hypoxic COPD patients, failure was considered as no, or a minimal, rise in PaO_2_/FiO_2_ after 1-2 hours (< 200) [13]. The Richmond agitation sedation scale (RASS) was used for infusion and assessment of the daily need for sedation [23]. When patients met the previously described criteria for weaning, they were extubated after 30 minutes of a successful T-piece trial [24]. After extubation, NIV was applied in cases of moderate respiratory distress, if there were no contraindications [24].

### Outcomes

The primary outcome was mortality (including hospital and 30 days after hospital discharge), and the secondary outcomes were NIV and IMV durations and the length of ICU stay.

### Statistical analysis

A descriptive analysis was used for patient demographics and ICU data. Case and control groups were compared with Mann Whitney U tests for non-parametric continuous variables or Student’s t-tests for parametric continuous variables. The chi-square test was employed for dichotomous variables. Median and quartiles 1 and 3 were used to describe non-parametric continuous variables, and mean ± standard deviation (SD) was used for parametric continuous variables. Count and percentage were used when applicable. For predicting the mortality rates of ICU, hospital, and 30 days after hospital discharge, logistic regression analysis was applied using a model that included HRLD type, APACHE II score on admission to the ICU, age, reasons for ICU admission, NIV and IMV implications, presence of septic shock, CRP level, and previous HRLD use. P < 0.05 was accepted as statistically significant.

## Results

**Figure 1:**
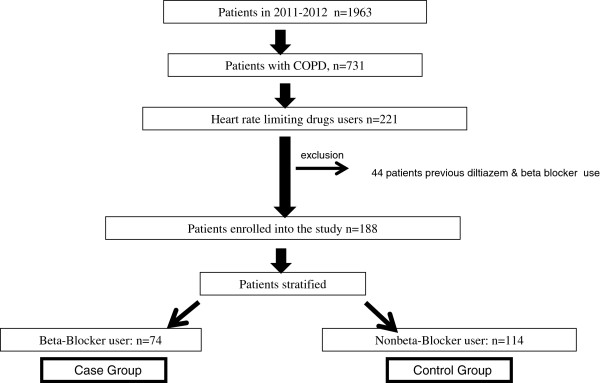
Flow chart of patients enrollment.

During the study period, a total of 1,964 patients were admitted to the ICU, and 221 patients were using HRLDs. After excluding 44 patients due to simultaneous beta-blocker and diltiazem use, 188 patients were included in the study. The patient enrollment flow chart is shown in Figure 1.

Patient group demographics, comorbidities, reason for ICU admission, microbiology results, and ICU data, including ABG analyses, are summarized in Table 1. The case group (using beta-blockers, n = 74) and the control group (using non-beta-blockers, n = 114) were not significantly different with regard to age, gender, body mass index, comorbidities, reason for ICU admission, APACHE II score, ABG on ICU admission, CRP values, or the presence of sepsis and septic shock (Table 1). Previous use of HRLDs before admission and in the ICU are shown in Table 2. Previous HRLD users in the case group were taking beta-blockers (27/31), and most in the control group were previously prescribed diltiazem (37/57). During the ICU stay, the case group received metoprolol, bisoprolol, or carvedilol, and those in control group received diltiazem, digoxin, amiodarone, and more than one HRLD (except beta-blockers), such as diltiazem plus amiodarone and/or plus digoxin (Table 2). SVT was the most common reason for HRLD use in both groups (Table 2). Heart rate and mean arterial pressure values on admission to the ICU and the highest values are also summarized in Table 2.

**Table 1:** Patient characteristics for groups (case: beta-blockers, control: non-beta-blockers)

	**Case, n = 74**	**Control, n = 114**	**p value**
Age, years, median (quartile 1–3)	71 (63–77)	71 (64–75)	0.82
Female/male	18/56	28/86	0.97
BMI, kg/m^2^, median (quartile 1–3)	23 (22–27)	24 (21–28)	0.82
**Comorbidities**, **n (%)**	69 (93.2)	100 (87.7)	0.22
AF, n (%)	56 (75.7)	80 (70.2)	0.41
Diabetes mellitus, n (%)	20 (27.0)	29 (25.4)	0.81
CAD, n (%)	12 (16.2)	14 (12.3)	0.45
Hypertension, n(%)	42(56.8)	73 (64)	0.32
***Pre ICU location, n (%)***			
Emergency service	40 (54.1)	55 (48.2)	0.69
Hospital ward	31 (41.9)	55 (48.2)	
Another ICU	3 (4.1)	4 (3.5)	
***Reason for ICU admission***			
COPD exacerbation	64 (86.5)	101 (88.6)	0.35
Pneumonia	8 (10.8)	6 (5.3)	
Hemodynamic monitoring	0 (0)	3 (2.6)	
Postoperative respiratory failure	0 (0)	1 (0.9)	
Home ventilator evaluation	2 (2.7)	0 (0)	
Sepsis, n (%)	50 (67.6)	76 (66.7)	0.90
Septic shock, n (%)	12 (16.2)	13 (11.4)	0.34
ICU admission APACHE-II value	19 (17–23)	20 (16–24)	0.88
ICU admission CRP mg/L	39 (14–91)	41 (14–125)	0.33
Peak of CRP mg/L median	64 (23–130)	110 (30–178)	0.08
Microbiologic culture, n (%)	51 (68.9)	67 (58.8)	0.16
Positive culture n (%)	18 (35.3)	27 (23.7)	0.58
Resistant pathogen, rate	14/18	23/27	0.43
***Arterial blood gases analysis on admission ICU***			
pH, median (quartile 1–3)	7.28 (7.25-7.37)	7.32 (7.25-7.40)	0.08
PaCO_2_, mmHg, median (quartile 1–3)	72 (54–86)	73 (59–84)	0.78
PaO_2_/FiO_2,_ median (quartile 1–3)	160 (130–211)	168 (116–230)	0.65
***Type of acute respiratory failure, n(%)***			
PaO_2_/FiO_2_ <300	15 (20.3)	19 (16.7)	0.50
PaO_2_/FiO_2_ < 300 and PaCO_2_ > 45 mmHg	46 (62.2)	79 (69.3)	
PaCO_2_ > 45 mmHg	13 (17.6)	16 (14.0)	

*AF,* atrial fibrillation; APACHEII*,* acute physiologic and chronic health evaluation II; *BMI*, body mass index; CAD*,* coronary artery disease; COPD, chronic obstructive pulmonary disease; CRP*,* C*-*reactive protein; *FiO*_*2*_, fractionated inspired oxygen; *ICU,* intensive care unit, *PaCO*_*2*_, partial arterial carbon dioxide pressure; *PaO*_*2*_*,* partial arterial oxygen pressure.

**Table 2:** Heart rate limiting drugs in groups (case: beta-blockers, control: non-beta-blockers)

	**Case, n = 74**	**Control, n = 114**	**p**
**Previous HRLD use, n (%)**	**31(41.9)**	**57 (50.0)**	**0.28**
***Previously used HRLDs***			
Beta-blocker, n (%)	27 (87.1)	4 (7.0)	0.001
Diltiazem, n (%)	1 (1.4)	37 (64.9)	
Amiodorone, n (%)	0 (0.0)	2 (3.5)	
Digitoxin, n (%)	0 (0.0)	3 (5.3)	
Multidrug*, n (%)	3 (12.9)	11 (19.3)	
***HRLDs used in ICU***			
***HRLD, n***	Metoprolol = 59	Diltiazem = 91	--
	Bisoprolol = 4	Digoxin = 2	
	Carvedilol = 11	Amiodorone = 3	
		*More than one =18	
***Reasons for HRLD use, n(%)***			
AF	17 (23.0)	35 (50.7)	0.004
SVT	31 (41.9)	62 (54.4)	
VT	0 (0.0)	1 (0.9)	
CHF	13 (17.6)	5 (4.4)	
HT	6 (8.1)	10 (8.8)	
Suspicion of MI	7 (9.5)	1 (0.9)	
**Only received HRLDs on day 1, n (%)**	4 (5.4)	13 (11.4)	0.16
**Intermittent HRLD use, n (%)**	7 (9.5)	11 (9.6)	0.97
**♦Heart rate/min on admission to ICU**	111 (21)	115 (25)	0.31
**♦Highest heart rate/min**	128 (25)	133 (20)	0.14
**♦MAP, mmHg on admission of ICU**	97 (26)	101 (25)	0.33
**♦Highest MAP, mmHg**	118 (20)	119 (21)	0.63

*More than one drug used (ie: diltiazem ± digitoxin ± amiadorone). *AF*, atrial fibrillation; *CHF,* congestive heart failure, *HRLD*, heart-rate limiting drug; *HT,* hypertension, ICU*,* intensive care unit; *MAP,* mean arterial pressure; *MI,* myocardial infarction, *SVT*, supraventricular tachycardia; *VT,* ventricular tachycardia. ^**♦**^mean (± standard deviation).

Types and duration of mechanical ventilation; lengths of ICU and hospital stays; ICU, hospital, and 30-day mortality after hospital discharge were compared between groups and shown in Table 3. The mortality rates in both groups with respect to ICU, hospital, and within 30 days of hospital discharge were similar. NIV application rate was significantly higher in beta-blocker users (Table 3).

**Table 3:** ICU outcome data of the two groups (case: beta-blockers, control: non-beta-blockers)

**Outcome**	**Case, n = 74**	**Control, n = 114**	**p**
Application of NIV, n (%)	67 (90.5)	87 (76.3)	0.013
NIV duration, day	6 (3–7)	5 (2–8)	0.84
Application of IMV, n (%)	25 (33.8)	40 (35.1)	0.85
IMV duration, day, median (quartile 1–3)	2 (1–5)	5 (2–9)	0.10
Length of ICU stay, median (quartile 1–3)	6 (4–10)	7 (4–10)	0.69
ICU mortality, %	17.6	15.8	0.75
Hospital mortality, %	18.9	19.3	0.95
30 day mortality, %	20.0 (12/60)	11.0 (10/91)	0.13

*ICU,* intensive care unit; *IMV,* Invasive mechanical ventilation, *NIV*, noninvasive mechanical ventilation.

**Figure 2:**
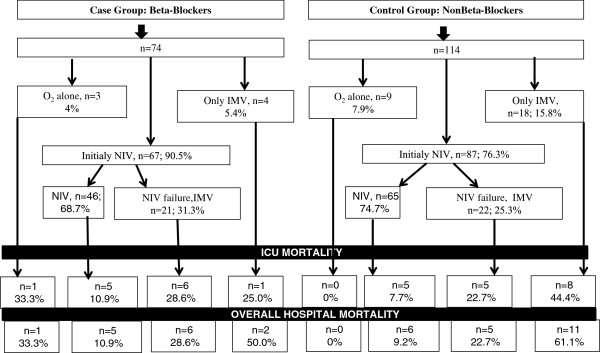
Mechanical ventilation and ICU mortality in study groups.

The initial mechanical ventilation (NIV or IMV) and ICU/hospital mortality outcomes in both groups are shown in Figure 2.

Eleven patients had do-not-intubate orders due to family request underlying the terminal status of the COPD (one patient had only nasal oxygen therapy, and 10 patients had only NIV treatment). In the only NIV-applied group who received beta-blockers, we noted an 11.1% mortality rate versus 7.8% with non-beta-blockers. However, all of the patients who died in the beta-blocker group (n = 6, 100%) died with resistant organisms compared to 60% of patients in the non-beta-blocker group who died with a resistant pathogen (n = 3). Five out of six (83.3%) patients in the beta-blocker group with failed NIV and who were intubated for IMV had septic shock due to a resistant pathogen.

A sub-analysis was performed in the case group to evaluate mortality related to previous beta-blocker use. There were 27 (36.5%) patients in the case group who had previously used beta-blockers (Table 2). When we compared them with patients who had only received beta-blockers in the ICU (n = 47) we found that 96.2% of the previous beta-blocker users required NIV versus 87.2% of the current beta-blocker users. Those who required IMV on admission to ICU were 0% in the previous beta-blocker users versus 8.5% in the current beta-blocker users, and those who required IMV after initially receiving NIV were 30.7% in the previous beta-blocker users versus 31.7% in the current beta-blocker users. These results were similar between the two groups. Mortality occurred in 4 of the 27 previous beta-blocker users (1 was receiving only medical treatment, 2 were on NIV only, and 1 received IMV after NIV failure). Nine out of the 47 current beta-blocker users died (1 had received only IMV, 2 received only NIV, and 5 received IMV after NIV failure). All five patients in whom NIV failed had a pan-resistant pathogen. CHF patients on beta-blockers before ICU admission (n = 13) or during the ICU stay (n = 11) had similar mortality rates (14.8% versus 19.1%, p = 0.64).

During the study period, 363/1,963 patients died (19.5%) in the ICU, and the mortality rate among the 731 patients with COPD who were admitted to the ICU was 13.8%. Among 188 COPD patients using HRLDs, the overall mortality rate was 16.5% (31/188).

For predicting ICU, hospital, and 30-day mortality rates after hospital discharge, we included APACHE II score on admission, age, reasons for ICU admission, NIV and IMV implications, presence of septic shock, CRP level, and previous HRLD use in the binary logistic regression model. The significant predictors and the effects of beta-blocker use are summarized in Table 4. Beta blocker use was not found to have a significant effect on mortality rate in the ICU, the hospital, or within 30 days of discharge. The presence of septic shock on admission to the ICU and lower PaO_2_/FiO_2_ were found to be significant risk factors for ICU mortality (Table 4). For hospital mortality, the presence of septic shock in the ICU, lower PaO_2_/FiO_2_, and HT were found to be associated with increased rate and conversely NIV application was with decreased rate (Table 4). The presence of coronary heart diseases increased the short-term (30 days) mortality risk after hospital discharge, whereas higher pH levels decreased the 30-day mortality rate (Table 4).

**Table 4:** Logistic regression analysis of mortality risk factors in the ICU, hospital and 30 days after hospital discharge

***ICU mortality risk factors***			
	**Odds ratio**	**Confidence interval 95%**	**p**
Septic shock in the ICU	7.49	2.43–23.14	0.001
PaO_2_/FiO_2_ on admission to the ICU	0.99	0.98–0.99	0.001
Beta-blocker use	1.14	0.52–2–49	0.75
***Hospital mortality risk factors***			
Septic shock in the ICU	6.29	2.03–19.49	0.001
Hypertension	2.94	1.05–8.23	0.040
NIV in the ICU	0.21	0.08–0.57	0.002
PaO_2_/FiO_2_ on admission to the ICU	0.99	0.98–0.99	0.010
Beta-blocker use	0.98	0.46–2.06	0.95
***30 days mortality risk factors***			
pH on admission to the ICU	0.71	0.63–0.79	0.001
Ischemic cardiac disease	4.77	1.49–15.24	0.009
Beta-blocker use	2.03	0.81–5.04	0.13

*FiO*_*2*_, fractionated inspired oxygen ratio; *ICU,* intensive care unit; *NIV,* noninvasive mechanical ventilation; *PaO*_*2*_*,* partial arterial oxygen pressure.

## Discussion

The present study showed similar mortality rates and length of ICU stay in COPD patients with ARF who received either beta-blockers or other HRLDs to control heart rate.

Although the useful effects of beta-blockers in the treatment of cardiac diseases are well-known, their use in COPD patients has been restricted due to possible contraindication [25]. It has been reported that selective and non-selective beta-blockers increase airway hyperresponsiveness (AHR) [9]. In a murine model of antigen-induced airway inflammation and AHR, acute and chronic treatment with beta-blockers increased and decreased AHR, respectively, but the mechanism of this event has not been established [26]. However, evidence from trials and meta-analyses suggests that cardioselective beta_1_-blockers should not be routinely withheld from patients with COPD because the potential benefits outweigh the risks [27]. A meta-analysis that pooled 22 randomized blinded control trials of patients with COPD demonstrated that cardioselective beta-blockers, given as a single dose or for longer durations, produced no significant change in FEV_1_ or respiratory symptoms [28]. In the present study, the rate of NIV application was significantly greater in beta-blocker users than in non-beta-blocker users, and the need of initial IMV was three times higher in the non-beta-blockers group, although that was not statistically significant. Notably beta-blocker use did not lead to a worsening in patient condition in our study.

Cardiovascular diseases (CVDs), including CAD, heart failure, AF, and HT are major comorbidities in COPD [7,29,30]. In the present study, AF was found to be the major comorbidity. Some short-term studies have demonstrated the safety of using selective beta-blockers in CAD with COPD [10,31]. Selective beta-blockers (e.g., bisoprolol) have a crucial effect on survival in patients with HF, but the presence of COPD is a common reason for patients not to receive sufficient therapy [7,32,33]. In the present study, the majority of patients were treated with metoprolol (79.7%), and a few were given bisoprolol (5%). Dransfield and coworkers reported that the prevalence of beta-blocker use in COPD patients with either MI or CHF was 31% [34]. However, there is no clear data regarding beta-blocker use in the ICU. The present study showed that beta-blockers were used for similar reasons in 19.4% of COPD patients. Some studies found that using cardioselective beta_1_-blockers in COPD patients with acute myocardial infarction was safe [10,11]. Conversely, other studies reported that beta-blockers diminished the beta-agonist effect [8,9]. Brooks and colleagues studied the rates of hospitalizations and emergency department (ED) visits during cardioselective and non-selective beta-blocker therapy in patients with asthma and/or COPD [35] and demonstrated the safety of cardioselective beta_1_-blockers in patients with COPD. Although the risk of ED visits was slightly increased, the risk of hospitalization was reduced. In the same study, non-selective beta-blocker therapy in COPD patients reduced both the rate of ED visits and the total number. These findings suggest a larger safety margin with beta-blocker therapy in patients with COPD compared to those with asthma, with or without COPD [35]. A recent study demonstrated the safety of beta-blocker treatment during COPD exacerbation in hospitalized patients with CVD [36]. The present findings also support the safety of beta-blockers compared with non-beta-blockers in COPD patients with ARF, due to the presence of CVD in the ICU.

Confalonieri and coworkers reported that the mortality rate of COPD patients with ARF in the ICU was 13.7%, and NIV and IMV failed in 60.2% and 49.2% of patients, respectively [37]. In this study, the overall mortality rate was 19.5%, and the mortality rate for COPD patients was 13.8%. However, the mortality rates for HRLD users, NIV failure patients, and initial IMV use in COPD patients were 16.5%, 25.6%, and 40.1%, respectively. Thus, our results are similar to those of Confalonieri et al. Although Alaithan and co-authors recently described a mortality rate in COPD patient populations as low as 6% in the ICU [38], the APACHE II scores were considerably lower than in our study and others.

There are some limitations in our study. Firstly, it was a retrospective, single-center study. A large, specific patient group followed by experienced ICU pulmonologists/intensivists could provide additional important results. Secondly, spirometry test scores were not recorded from the patients’ files. Thirdly, these findings are relevant to a specific patient population and cannot be generalized for all patients. The mortality rates of the groups were very close to each other and although a reasonable number of patients was included in our study, the sample size was not large enough to show a significant difference.

We found that a large number of COPD patient using HRLDs (either beta-blockers or non-beta-blockers) had similar outcomes (mortality and length of ICU stay). However, these patients had a higher rate (16.5%) of mortality than COPD patients who were not treated with HRLDs in the ICU (13.8%). The ~33% increase in mortality among HRLD users can be explained by the primary reason that these drugs are administered in the ICU. Severe sepsis, unresponsiveness to treatment, and septic shock were the main mortality risk factors in our patient population.

## Conclusions

This study provides a contribution to the controversial topic of using beta-blockers to limit heart rate in COPD patients with ARF in the ICU. As with other HRLDs, beta-blockers are utilizable for patients with bronchoconstriction due to underlying COPD in the ICU.

## Competing interest

The authors declare that they have no competing interests.
